# Comparison of postoperative outcomes following endoscopic third ventriculostomy or shunt in a propensity score matched pediatric cohort

**DOI:** 10.1007/s00381-025-06886-2

**Published:** 2025-07-31

**Authors:** Paulo Castro, Loren Berman, Joseph Piatt

**Affiliations:** 1 Department of Surgery, UConn Health, Farmington, CT USA; 2 Division of Pediatric Surgery, Nemours Children’s Health, Wilmington, DE USA; 3 Department of General Surgery, Thomas Jefferson University Hospital, Philadelphia, PA USA; 4 Department of Pediatrics, Sidney Kimmel Medical College, Thomas Jefferson University, Philadelphia, PA USA; 5 Division of Neurosurgery, Nemours Children’s Health, 1600 Rockland Road, Wilmington, DE 19803 USA; 6 Department of Neurological Surgery, Sidney Kimmel Medical College, Thomas Jefferson University, Philadelphia, PA USA

**Keywords:** Endoscopic third ventriculostomy, Hydrocephalus, National Surgical Quality Improvement Program, Outcomes

## Abstract

**Purpose:**

Thirty-day readmission and reoperation are widely used quality metrics. Endoscopic third ventriculostomy (ETV) is favored when feasible, but may compare unfavorably with shunt in the short-term. This study analyzes 30-day outcomes of ETV and shunt surgery in comparable populations.

**Methods:**

Data regarding patients undergoing ETV or initial shunt insertion were extracted from the National Surgical Quality Improvement Program – Pediatric for 2013–2020. Patients were stratified into three age groups: ≤ 6 months (N = 5,906), 6 months-2 years (N = 2,364), and ≥ 2 years (N = 4,408). Characteristics were compared before and after propensity-score matching. Outcome comparisons included CNS complications, mortality, readmission, and reinterventions.

**Results:**

There were 10,135 shunt insertions and 2,543 ETVs. After matching, patients ≤ 6 months undergoing ETV had more seizures (ETV 6.3% vs shunt 0.4%, p < 0.001), readmission (ETV 15.7% vs shunt 6.1%, p < 0.001), and reintervention (ETV 17.4% vs shunt 4.8%, p < 0.001). Among patients 6 months—2 years, ETV increased seizures (ETV 3.3% vs shunt 1.0%, p = 0.01), readmission (ETV 14.9% vs shunt 7.8%, p < 0.001), and reintervention (ETV 13.0% vs shunt 5.4%, p < 0.001). Among older patients, ETV had decreased median length of stay (ETV 3 days, IQR 1–6 days vs shunt 3 days, IQR 2–8 days, p = 0.0019) and mortality (ETV 0.4% vs shunt 1.5%, p = 0.007).

**Conclusion:**

Outcomes following shunt surgery and ETV in matched patients appear to be age-dependent. Younger patients undergoing ETV encountered more short-term complications. 30-day outcomes may be misleading as a quality measure in the management of childhood hydrocephalus.

## Introduction

Hydrocephalus is the most common condition requiring intervention in pediatric neurosurgery. In contemporary practice, there are only two treatment options: shunt insertion or endoscopic third ventriculostomy (ETV). Current neurosurgical practice favors ETV whenever it is feasible as this intervention avoids the issues associated with shunt dependence in the long-term; however, like shunts, ETV may fail and require reintervention in the short-term [1–24]. The relative benefit of each procedure for overall quality of life is still not clear [1–10]. A recent study of 30-day outcomes of more than 29,000 procedures found that, relative to new shunt placement, ETV led to significantly higher rates of unplanned return to the operating room [25]. This observation suggests that the decision to pursue the long-term benefits of ETV may be penalized by greater risk of short-term complications, but there were significant baseline differences in the shunt and ETV groups that might have driven the differences in outcomes. There are currently no reports in the literature comparing short-term outcomes for cases in which equipoise exists, that is, treatment by either procedure is clinically suitable. Success rates of ETV depend on many clinical factors, including patient age, etiology of hydrocephalus, and imaging findings [23]. In practice, ETV is the clear standard of care for some cases, while for others it may be relatively contraindicated. We hypothesized that, when there is a reasonable clinical choice between ETV and shunt insertion, pursuit of the long-term benefits of successful ETV may incur a cost of more frequent short-term complications. The current study refines the comparison of 30-day outcomes following ETV and shunt insertion in a large propensity score matched pediatric cohort.

## Methods

### Data and study population

We performed a retrospective review of the American College of Surgeons National Surgical Quality Improvement Program-Pediatric (NSQIP-P) database from 2013–2020. The NSQIP-P database includes children undergoing surgery up to age 18 in over 130 North American hospitals. The unit of observation is the index surgical procedure. (Clinical events prior to the index procedure are not captured.) The registry records patient characteristics, comorbidities, preoperative and intraoperative details, and 30-day postoperative outcomes including mortality. Readmissions with 30 days of the index procedure are identified as related or unrelated. Data are extracted from the clinical record by trained surgical clinical reviewers at each site using uniform definitions of conditions and outcomes. The data are deidentified with respect both to the patient and the institution.

The Nemours Institutional Review Board judged this project not to be human subjects research.

We identified all cases of endoscopic third ventriculostomy (ETV) and new shunt insertion during the study period. ETV was identified by Current Procedural Terminology (CPT) codes 62,200 and 62,201. New shunt placement was identified by CPT codes 62,220 and 62,223. Cases were separated into three different age groups: ≤ 6 months, 6 months to 2 years, and ≥ 2 years based on the senior author’s perception of surgical challenges.

### Primary characteristics and outcomes

Extracted demographic and clinical data included age in days at the time of surgery, weight at time of surgery, gestational age at birth, history of prematurity (defined as < 37 weeks gestational age at birth), sex, race, and American Society of Anesthesiology (ASA) classification. Race was dichotomized into White and non-White (other and unknown race were included in this category) given the low counts for certain categories within this variable. The comorbidities and pertinent preoperative clinical factors extracted included ventilator dependence, cardiac risk factors (none, minor, major, and severe), intraventricular hemorrhage (IVH), steroid use within 30 days preoperatively, hematologic disorder, history of cardiac surgery, case status (elective, urgent, emergent), oxygen support, developmental delay, seizure disorder, cerebral palsy, structural CNS abnormality, neuromuscular disorder, ostomy, nutritional support, SIRS/sepsis/septic shock within 48 h prior to surgery, inotropic support, history of CPR within 7 days prior to surgery, congenital malformation, childhood malignancy, and blood transfusion within 48 h preoperatively. These variables were collected to evaluate baseline differences between the two groups.

The primary outcomes were the incidence of 30-day reintervention or readmission related to the index procedure. Other outcomes of interest were operative time, length of stay, and postoperative complications such as: surgical site infection (SSI) (superficial, deep, and organ space), wound disruption, sepsis, septic shock, unplanned intubation, mechanical ventilation for > 48 h, pneumonia, blood transfusion volume of 25 mL/kg intra-operatively or in the first 72 h after surgery, acute renal failure, progressive renal insufficiency, cardiac arrest, ventricular tachycardia, coma, stroke, seizure, nerve injury, IVH, urinary tract infection, central line infection, continuing hospitalization at 30 days, and death.

### Statistical analysis

All numeric variables were reported as a median with an interquartile range (IQR) and compared using a Mann–Whitney U test. Pearson’s chi-square or Fisher’s exact tests, where appropriate, were used for categorical variables. All statistical tests were 2-sided. A p-value < 0.05 was considered statistically significant. Descriptive and comparative statistics of preoperative variables were performed prior to matching. A logistic regression model was used to calculate propensity scores using all preoperative characteristics available, as listed previously. A propensity score match was then completed between ETV and shunt groups in a 1-to-1 fashion without replacement using the caliper-matching method. The propensity score caliper was determined by multiplying the standard deviation of the logit of propensity scores by 0.2. Within the matched cohort, descriptive and comparative statistics of the preoperative variables and postoperative outcomes were performed between groups. Pre- and post-match standardized percent biases were compared across all covariates. Readmission survival analysis was performed using a log-rank test. Variables with missing data were reported with the true count in parentheses beside the variable name in the data tables. A complete case analysis was performed. This analysis was completed utilizing STATA/SE 15.1 statistical software (StataCorp LLC, College Station, TX).

The STrengthening the Reporting of OBservational studies in Epidemiology (STROBE) checklist was followed.

## Results

### Study population

Prior to matching, there were a total of 10,135 shunt insertions and 2,543 ETVs performed. In the ≤ 6 months age group, there were 5,906 cases including 5,328 shunts and 578 ETVs. In the 6 months to 2 years age group, there were 2,364 cases including 1,792 shunts and 572 ETVs. In the ≥ 2 years age group, there were 4,408 cases comprised of 3,015 shunts and 1,393 ETVs.

In the ≤ 6 months age group, the ETV patients were older (85 vs 60 days, p < 0.001), heavier (4.8 vs 3.6 kgs, p < 0.001), more likely to be born full term (58.1% vs 47.2%, < 0.001), less likely to be ventilator dependent (6.9% vs 14.0%, p < 0.001), more likely to have no CRFs (65.6% vs 59.6%, p = 0.04), less likely to have a hematologic disorder (12.1% vs 19.8%, p < 0.001), less likely to require oxygen support (13.7% vs 24.7%, p < 0.001), less likely to have a history of seizure (11.6% vs 15.4%, p = 0.01), more likely to have a structural CNS abnormality (92.9%, vs 89.5%, p = 0.01), more likely to have a neuromuscular disorder (8.5% vs 5.5%, p < 0.01), more likely to not have history of IVH (71.5% vs 56.4%, p < 0.001), less likely to require nutritional support (22.3% vs 36.0%, p < 0.001), and more likely to have a congenital malformation (68.9% vs 63.3%, p = 0.01) compared to new shunt patients. Choroid plexus cauterization is often performed in conjunction with ETV in this age group, but in the absence of a specific CPT code confident identification of this procedure was impossible.

In the 6 months to 2 years age group, the ETV patients were older (320 vs 307 days, p = 0.04), heavier (9.2 vs 8.6 kgs, p < 0.0001), less likely to have a history of seizure (11.4% vs 21.0%, p < 0.001), more likely to have a structural CNS abnormality (93.4% vs 89.3%, p = 0.004), less likely to have prior ostomy (14.4% vs 19.7%, p < 0.001), less likely to require nutritional support (12.8% vs 20.8%, p < 0.001), and more likely to have a congenital malformation (65.2% vs 58.6%, p = 0.005) compared to new shunt patients.

In the ≥ 2 years age group, the ETV patients were older (3784 vs 3166 days, p < 0.0001), heavier (37 vs 28 kgs, p < 0.0001), more likely to be born full term (83.6% vs 78.9%, p < 0.001), less likely to be ventilator dependent (1.7% vs 5.1%, p < 0.001), more likely to have no CRFs (94.1% vs 89.4%, p < 0.001), less likely to have needed steroids within 30 days (10.4% vs 16.7%, p < 0.001), less likely to have a hematologic disorder (2.9% vs 6.3%, p < 0.001), less likely to have previous cardiac surgery (2.4% vs 3.7%, p < 0.03), less likely to require oxygen support (1.8% vs 5.5%, p < 0.001), less likely to have developmental delay (27.1% vs 37.9%, p < 0.001), less likely to have a history of seizure (11.8% vs 23.2%, p < 0.001), less likely to have cerebral palsy (5.8% vs 10.5%, p < 0.001), less likely to have a neuromuscular disorder (8.6% vs 15.6%, p < 0.001), more likely to not have history of IVH (94.1% vs 87.6%, p < 0.001), less likely to have prior ostomy (7.1% vs 18.4%, p < 0.001), less likely to require nutritional support (3.9% vs 15.6%, p < 0.001), and less likely to have a congenital malformation (31.5% vs 37.7%, p < 0.001) compared to new shunt patients. The preoperative characteristics of these patients are shown in Table 1.

**Table 1:** Pre-match preoperative characteristics

Variable		New Shunt	ETV	p-value	New Shunt	ETV	p-value	New Shunt	ETV	p-value
		≤ 6 months			6 months—2 years			≥ 2 years		
Sex	Male	2958 (55.5%)	327 (56.6%)	0.627	1005 (56.1%)	334 (58.4%)	0.332	1665 (55.2%)	766 (55.0%)	0.784
	Female	2370 (44.5%)	251 (43.4%)		787 (43.9%)	238 (41.6%)		1349 (44.7%)	627 (45.0%)	
	Non-Binary	0 (0.0%)	0 (0.0%)		0 (0.0%)	0 (0.0%)		1 (0.0%)	0 (0.0%)	
Race	White	3017 (56.6%)	345 (59.7%)	0.158	1062 (59.3%)	354 (61.9%)	0.265	1966 (65.2%)	881 (63.2%)	0.205
	Non-White	2311 (43.4%)	233 (40.3%)		730 (40.7%)	218 (38.1%)		1049 (34.8%)	512 (36.8%)	
Age at Surgery, days (IQR)		60.0 (21.0–102.0)	85.0 (36.0–132.3)	** < 0.0001**	307.0 (229.0–443.8)	319.5 (247.5–452.0)	**0.0358**	3166.0 (1724.0–4906.0)	3784.0 (2157.0–5177.0)	** < 0.0001**
Weight at Surgery, kgs (IQR) (n = 28,969)		3.6 (2.8–4.9)	4.8 (3.5–6.3)	** < 0.0001**	8.6 (7.3–10.3%)	9.2 (7.8–10.8)	** < 0.0001**	28.0 (17.2–49.2)	37.0 (21.0–56.9)	** < 0.0001**
Gestational Age at Birth (n = 7,753)	< = 24	512 (9.6%)	40 (7.0%)	** < 0.001**	167 (9.5%)	37 (6.6%)	0.733	98 (3.7%)	15 (1.3%)	** < 0.001**
	25–26	543 (10.2%)	24 (4.2%)		119 (6.8%)	40 (7.1%)		106 (4.0%)	34 (2.9%)	
	27–28	408 (7.7%)	17 (3.0%)		99 (5.6%)	31 (5.5%)		80 (3.0%)	27 (2.3%)	
	29–30	239 (4.5%)	23 (4.0%)		54 (3.1%)	15 (2.7%)		44 (1.6%)	16 (1.4%)	
	31–32	247 (4.7%)	19 (3.3%)		63 (3.6%)	20 (3.6%)		53 (2.0%)	27 (2.3%)	
	33–34	373 (7.0%)	45 (7.9%)		135 (7.7%)	42 (7.5%)		67 (2.5%)	26 (2.2%)	
	35–36	482 (9.1%)	71 (12.4%)		163 (9.3%)	53 (9.4%)		117 (4.4%)	49 (4.1%)	
	Full term	2502 (47.2%)	332 (58.1%)		956 (54.4%)	323 (57.6%)		2109 (78.9%)	988 (83.6%)	
Prematurity (n = 27,343)		2804 (52.8%)	239 (41.9%)	** < 0.001**	800 (45.6%)	238 (42.4%)	0.194	565 (21.1%)	192 (16.4%)	**0.001**
Ventilator Dependence		748 (14.0%)	40 (6.9%)	** < 0.001**	88 (4.9%)	28 (4.9%)	0.988	155 (5.1%)	23 (1.7%)	** < 0.001**
Cardiac Risk Factors	Major	1076 (20.2%)	97 (16.8%)	**0.044**	200 (11.2%)	54 (9.4%)	0.401	125 (4.1%)	39 (2.8%)	** < 0.001**
	Minor	1037 (19.5%)	99 (17.1%)		272 (15.2%)	93 (16.3%)		185 (6.1%)	40 (2.9%)	
	None	3174 (59.6%)	379 (65.6%)		1301 (72.6%)	422 (73.8%)		2695 (89.4%)	1311 (94.1%)	
	Severe	41 (0.8%)	3 (0.5%)		19 (1.1%)	3 (0.5%)		10 (0.3%)	3 (0.2%)	
Steroid Use within 30 days		336 (6.3%)	30 (5.2%)	0.291	110 (6.1%)	27 (4.7%)	0.206	502 (16.7%)	145 (10.4%)	** < 0.001**
Hematologic Disorder		1056 (19.8%)	70 (12.1%)	** < 0.001**	148 (8.3%)	37 (6.5%)	0.165	191 (6.3%)	41 (2.9%)	** < 0.001**
Previous Cardiac Surgery		313 (5.9%)	28 (4.8%)	0.313	127 (7.1%)	40 (7.0%)	0.939	112 (3.7%)	34 (2.4%)	**0.028**
Case Status	Elective	3833 (71.9%)	443 (76.6%)	0.056	1370 (76.5%)	451 (78.8%)	0.099	1935 (64.2%)	858 (61.6%)	0.235
	Emergent	521 (9.8%)	47 (8.1%)		204 (11.4%)	47 (8.2%)		538 (17.8%)	261 (18.7%)	
	Urgent	974 (18.3%)	88 (15.2%)		218 (12.2%)	74 (12.9%)		542 (18.0%)	274 (19.7%)	
ASA Class (n = 19,435)	1	19 (0.6%)	1 (0.3%)	**0.008**	6 (0.5%)	3 (0.9%)	0.294	9 (0.5%)	15 (1.8%)	** < 0.001**
	2	525 (15.7%)	69 (21.7%)		271 (24.0%)	95 (28.7%)		395 (20.7%)	277 (32.8%)	
	3	2287 (68.2%)	217 (68.2%)		767 (68.1%)	209 (63.1%)		1327 (69.5%)	509 (60.3%)	
	4	514 (15.3%)	31 (9.7%)		83 (7.4%)	24 (7.3%)		172 (9.0%)	42 (5.0%)	
	5	9 (0.3%)	0 (0.0%)		0 (0.0%)	0 (0.0%)		7 (0.4%)	1 (0.1%)	
Oxygen Support		1316 (24.7%)	79 (13.7%)	** < 0.001**	189 (10.5%)	56 (9.8%)	0.605	167 (5.5%)	25 (1.8%)	** < 0.001**
Developmental delay/impaired cognitive status		674 (12.7%)	75 (13.0%)	0.823	842 (47.0%)	268 (46.9%)	0.956	1142 (37.9%)	377 (27.1%)	** < 0.001**
Seizure Disorder		822 (15.4%)	67 (11.6%)	**0.014**	376 (21.0%)	65 (11.4%)	** < 0.001**	698 (23.2%)	165 (11.8%)	** < 0.001**
Cerebral Palsy		22 (0.4%)	1 (0.2%)	0.379	58 (3.2%)	13 (2.3%)	0.24	318 (10.5%)	81 (5.8%)	** < 0.001**
Structural CNS Abnormality		4766 (89.5%)	537 (92.9%)	**0.009**	1600 (89.3%)	534 (93.4%)	**0.004**	2563 (85.0%)	1174 (84.3%)	0.531
Neuromuscular Disorder		292 (5.5%)	49 (8.5%)	**0.003**	215 (12.0%)	59 (10.3%)	0.274	471 (15.6%)	120 (8.6%)	** < 0.001**
Intraventricular Hemorrhage	Grade 1	70 (1.3%)	6 (1.0%)	** < 0.001**	18 (1.0%)	3 (0.5%)	0.131	7 (0.2%)	3 (0.2%)	** < 0.001**
	Grade 2	113 (2.1%)	8 (1.4%)		22 (1.2%)	12 (2.1%)		17 (0.6%)	6 (0.4%)	
	Grade 3	592 (11.1%)	33 (5.7%)		115 (6.4%)	25 (4.4)		56 (1.9%)	15 (1.1%)	
	Grade 4	1195 (22.4%)	86 (14.9%)		199 (11.1%)	61 (10.7%)		119 (3.9%)	24 (1.7%)	
	Unknown Grade	354 (6.6%)	32 (5.5%)		101 (5.6%)	25 (4.4)		174 (5.8%)	34 (2.4%)	
	None	3004 (56.4%)	413 (71.5%)		1337 (74.6%)	446 (78.0%)		2642 (87.6%)	1311 (94.1%)	
Ostomy (n = 23,335)		321 (6.3%)	38 (7.1%)	0.879	338 (19.7%)	78 (14.4%)	** < 0.001**	536 (18.4%)	94 (7.1%)	** < 0.001**
Nutritional support		1918 (36.0%)	129 (22.3%)	** < 0.001**	372 (20.8%)	73 (12.8%)	** < 0.001**	469 (15.6%)	54 (3.9%)	** < 0.001**
SIRS/Sepsis/Septic Shock within 48 h prior	None	5239 (98.3%)	572 (99.0%)	0.514	1744 (97.3%)	559 (97.7%)	0.379	2856 (94.7%)	1338 (96.1%)	0.181
	Sepsis	17 (0.3%)	1 (0.2%)		7 (0.4%)	4 (0.7%)		23 (0.8%)	5 (0.4%)	
	Septic Shock	0 (0.0%)	0 (0.0%)		0 (0.0%)	0 (0.0%)		1 (0.0%)	0 (0.0%)	
	SIRS	72 (1.4%)	5 (0.9%)		41 (2.3%)	9 (1.6%)		135 (4.5%)	50 (3.6%)	
Inotropic support at time of surgery		21 (0.4%)	3 (0.5%)	0.654	5 (0.3%)	1 (0.2%)	0.666	10 (0.3%)	7 (0.5%)	0.395
Previous CPR within 7 days prior to surgery		17 (0.3%)	1 (0.2%)	0.545	2 (0.1%)	2 (0.3%)	0.228	4 (0.1%)	3 (0.2%)	0.522
Congenital Malformation		3373 (63.3%)	398 (68.9%)	**0.008**	1051 (58.6%)	373 (65.2%)	**0.005**	1138 (37.7%)	439 (31.5%)	** < 0.001**
Childhood Malignancy	No	5275 (99.0%)	575 (99.5%)	0.488	1656 (92.4%)	545 (95.3%)	0.062	2088 (69.3%)	969 (69.6%)	0.938
	Past Hx	5 (0.1%)	0 (0.0%)		5 (0.3%)	1 (0.2%)		81 (2.7%)	35 (2.5%)	
	Current	48 (0.9%)	3 (0.5%)		131 (7.3%)	26 (4.5%)		846 (28.1%)	389 (27.9%)	

Bold p-value indicates significant result at p < 0.05

*ETV* endoscopic third ventriculostomy, *IQR* interquartile range, *ASA* American Society of Anesthesiology.

Analysis comparing preoperative characteristics of patients who underwent endoscopic third ventriculostomy or new shunt insertion before propensity score matching

### Propensity score match

**Fig. 1:**
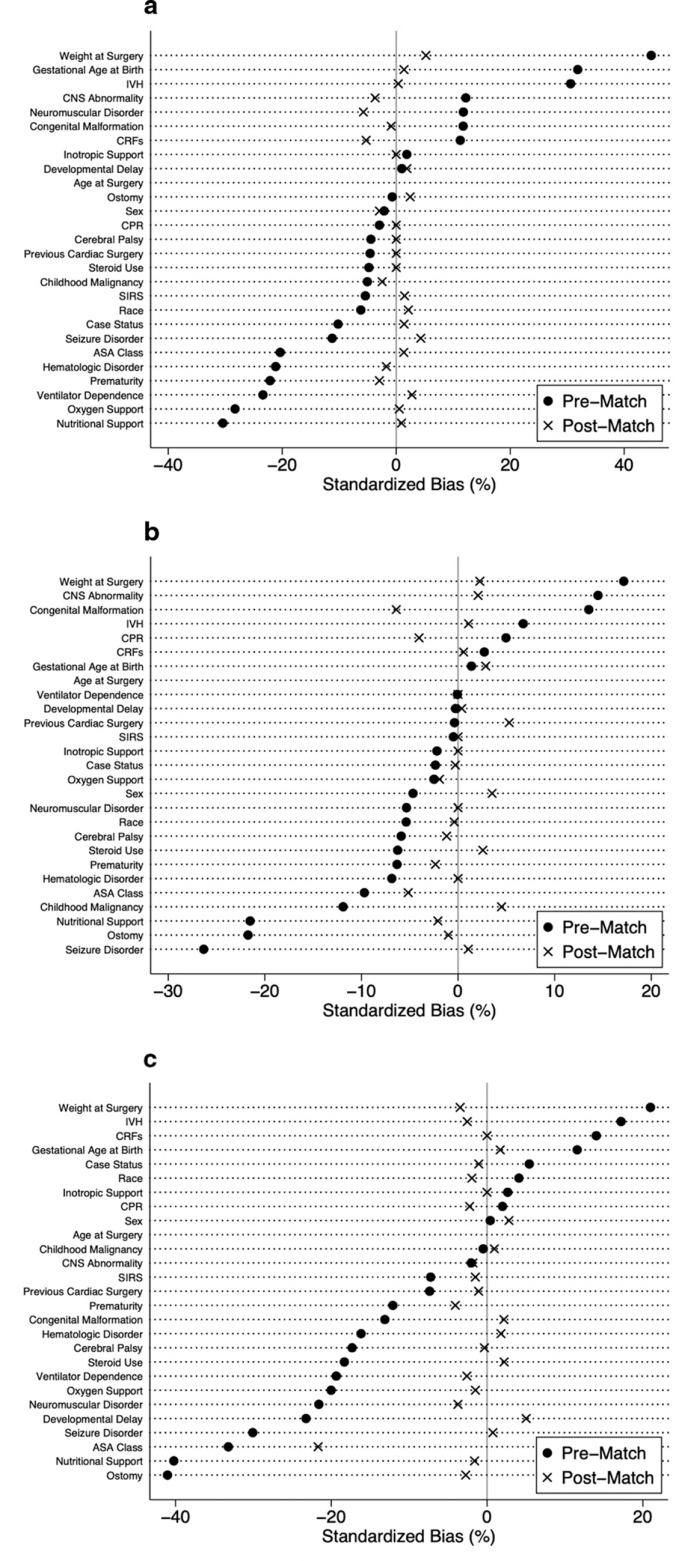
Covariate bias. Analysis comparing the percent standardized bias of pre- and post-match preoperative characteristics for the each age group: (**a**) ≤ 6 months, (**b**) 6 months – 2 years, (**c**) ≤ 6 months

Post-match, all patient characteristics in each age group were similar with the exception of ASA Class (p = 0.002) in the ≥ 2 years age group (Table 2). The quality of the match was assessed using pre- and post-match standardized percent bias for each covariate in each age group (Fig. 1).

**Table 2:** Post-match preoperative characteristics

Variable		New Shunt	ETV	p-value	New Shunt	ETV	p-value	New Shunt	ETV	p-value
		≤ 6 months			6 months—2 years			≥ 2 years		
Sex	Male	271 (56.8%)	278 (58.3%)	0.647	208 (40.3%)	217 (42.1%)	0.569	596 (55.7%)	580 (54.2%)	0.468
	Female	206 (43.2%)	199 (41.7%)		208 (40.3%)	217 (42.1%)		474 (44.3%)	491 (45.9%)	
	Non-Binary	0 (0.0%)	0 (0.0%)		0 (0.0%)	0 (0.0%)		1 (0.1%)	0 (0.0%)	
Race	White	300 (62.9%)	295 (61.8%)	0.738	319 (61.8%)	320 (62.0%)	0.949	700 (65.4%)	710 (66.3%)	0.649
	Non-White	177 (37.1%)	182 (38.2%)		197 (38.2%)	196 (38.0%)		371 (34.6%)	361 (33.7%)	
Age at Surgery, days (IQR)		87 (35–128)	85 (36–134)	0.497	311 (230–462)	314 (247–448)	0.527	3579 (2084–5175)	3540 (1946–4963)	0.183
Weight at Surgery, kgs (IQR)		4.7 (3.4–6.3)	4.9 (3.6–6.3)	0.123	9.0 (7.6–10.9)	9.2 (7.7–10.8)	0.732	34.2 (20.0–54.5)	34.7 (19.7–54.2)	0.785
Gestational Age at Birth	< = 24	32 (6.7%)	21 (4.4%)	0.707	27 (5.2%)	31 (6.0%)	0.922	21 (2.0%)	12 (1.1%)	0.802
	25–26	19 (4.0%)	15 (3.1%)		33 (6.4%)	36 (7.0%)		30 (2.8%)	29 (2.7%)	
	27–28	14 (2.9%)	11 (2.3%)		37 (7.2%)	30 (5.8%)		26 (2.4%)	26 (2.4%)	
	29–30	11 (2.3%)	17 (3.6%)		14 (2.7%)	13 (2.5%)		18 (1.7%)	15 (1.4%)	
	31–32	17 (3.6%)	17 (3.6%)		22 (4.3%)	18 (3.5%)		23 (2.2%)	26 (2.4%)	
	33–34	38 (8.0%)	40 (8.4%)		35 (6.8%)	41 (8.0%)		24 (2.2%)	25 (2.3%)	
	35–36	54 (11.3%)	57 (12.0%)		57 (11.1%)	50 (9.7%)		50 (4.7%)	42 (3.9%)	
	Full term	292 (61.2%)	299 (62.7%)		291 (56.4%)	297 (57.6%)		879 (82.1%)	896 (83.7%)	
Prematurity		185 (38.8%)	178 (37.3%)	0.641	225 (43.6%)	219 (42.4%)	0.706	192 (17.9%)	175 (16.3%)	0.33
Ventilator Dependence		18 (3.8%)	22 (4.6%)	0.518	21 (4.1%)	21 (4.1%)	1.0	24 (2.2%)	19 (1.8%)	0.441
Cardiac Risk Factors	Major	65 (13.6%)	71 (14.9%)	0.646	56 (10.9%)	48 (9.3%)	0.447	27 (2.5%)	31 (2.9%)	0.659
	Minor	71 (14.9%)	77 (16.1%)		74 (14.3%)	85 (16.5%)		46 (4.3%)	37 (3.5%)	
	None	338 (70.9%)	328 (68.8%)		381 (73.8%)	381 (73.8%)		996 (93.0%)	1002 (93.6%)	
	Severe	3 (0.6%)	1 (0.2%)		5 (1.0%)	2 (0.4%)		2 (0.2%)	1 (0.1%)	
Steroid Use within 30 days		22 (4.6%)	22 (4.6%)	1.0	19 (3.7%)	22 (4.3%)	0.633	98 (9.2%)	106 (9.9%)	0.556
Hematologic Disorder		48 (10.1%)	45 (9.4%)	0.743	33 (6.4%)	33 (6.4%)	1.0	26 (2.4%)	30 (2.8%)	0.588
Previous Cardiac Surgery		17 (3.6%)	17 (3.6%)	1.0	24 (4.7%)	31 (6.0%)	0.332	29 (2.7%)	27 (2.5%)	0.787
Case Status	Elective	368 (77.2%)	369 (77.4%)	0.647	404 (78.3%)	409 (79.3%)	0.612	674 (62.9%)	683 (63.8%)	0.875
	Emergent	44 (9.2%)	37 (7.8%)		54 (10.5%)	45 (8.7%)		199 (18.6%)	190 (17.7%)	
	Urgent	65 (13.6%)	71 (14.9%)		58 (11.2%)	62 (12.0%)		198 (18.5%)	198 (18.5%)	
ASA Class (n = 2,350)	1	0 (0.0%)	1 (0.2%)	0.429	3 (1.0%)	2 (0.7%)	0.83	4 (0.7%)	8 (1.3%)	**0.002**
	2	71 (24.5%)	55 (20.7%)		72 (24.2%)	79 (27.3%)		138 (23.0%)	200 (32.9%)	
	3	185 (63.8%)	184 (69.2%)		199 (67.0%)	187 (64.7%)		424 (70.7%)	373 (61.4%)	
	4	33 (11.4%)	26 (9.8%)		23 (7.7%)	21 (7.3%)		32 (5.3%)	26 (4.3%)	
	5	1 (0.3%)	0 (0.0%)		0 (0.0%)	0 (0.0%)		2 (0.3%)	1 (0.2%)	
Oxygen Support		48 (10.1%)	49 (10.3%)	0.915	49 (9.5%)	46 (8.9%)	0.747	25 (2.3%)	22 (2.1%)	0.658
Developmental delay/impaired cognitive status		57 (12.0%)	60 (12.6%)	0.767	241 (46.7%)	242 (46.9%)	0.95	281 (26.2%)	306 (28.6%)	0.226
Seizure Disorder		39 (8.2%)	46 (9.6%)	0.426	58 (11.2%)	60 (11.6%)	0.845	131 (12.2%)	134 (12.5%)	0.844
Cerebral Palsy		1 (0.2%)	1 (0.2%)	1.0	12 (2.3%)	11 (2.1%)	0.833	71 (6.6%)	70 (6.5%)	0.931
Structural CNS Abnormality		449 (94.1%)	444 (93.1%)	0.508	483 (93.6%)	486 (94.2%)	0.696	914 (85.3%)	907 (84.7%)	0.672
Neuromuscular Disorder		51 (10.7%)	44 (9.2%)	0.449	55 (10.7%)	55 (10.7%)	1.0	111 (10.4%)	98 (9.2%)	0.344
Intraventricular Hemorrhage	Grade 1	3 (0.6%)	5 (1.1%)	0.896	5 (1.0%)	3 (0.6%)	0.521	0 (0.0%)	2 (0.2%)	0.067
	Grade 2	7 (1.5%)	5 (1.1%)		5 (1.0%)	11 (2.1%)		2 (0.2%)	6 (0.6%)	
	Grade 3	29 (6.1%)	27 (5.7%)		28 (5.4%)	22 (4.3%)		11 (1.0%)	14 (1.3%)	
	Grade 4	50 (10.5%)	54 (11.3%)		50 (9.7%)	52 (10.1%)		21 (2.0%)	22 (2.1%)	
	Unknown Grade	35 (7.3%)	29 (6.1%)		31 (6.0%)	25 (4.8%)		44 (4.1%)	24 (2.2%)	
	None	353 (74.0%)	357 (74.8%)		397 (76.9%)	403 (78.1%)		993 (92.7%)	24 (2.2%)	
Ostomy		26 (5.5%)	29 (6.1%)	0.677	76 (14.7%)	74 (14.3%)	0.86	92 (8.6%)	82 (7.7%)	0.429
Nutritional support		81 (17.0%)	83 (17.4%)	0.864	62 (12.0%)	58 (11.2%)	0.698	47 (4.4%)	42 (3.9%)	0.588
SIRS/Sepsis/Septic Shock within 48 h prior	None	2 (0.4%)	3 (0.6%)	0.654	504 (97.7%)	505 (97.9%)	0.821	1037 (96.8%)	1041 (97.2%)	0.872
	Sepsis	0 (0.0%)	0 (0.0%)		3 (0.6%)	4 (0.8%)		2 (0.2%)	2 (0.2%)	
	Septic Shock	0 (0.0%)	0 (0.0%)		0 (0.0%)	0 (0.0%)		0 (0.0%)	0 (0.0%)	
	SIRS	2 (0.4%)	3 (0.6%)		9 (1.7%)	7 (1.4%)		32 (3.0%)	28 (2.6%)	
Inotropic support at time of surgery		2 (0.4%)	2 (0.4%)	1.0	1 (0.2%)	1 (0.2%)	1.0	5 (0.5%)	5 (0.5%)	1.0
Previous CPR within 7 days prior to surgery		No cases	No cases		1 (0.2%)	0 (0.0%)	0.317	2 (0.2%)	1 (0.1%)	0.563
Congenital Malformation		335 (70.2%)	333 (69.8%)	0.888	353 (68.4%)	337 (65.3%)	0.29	357 (33.3%)	368 (34.4%)	0.615
Childhood Malignancy	No	474 (99.4%)	475 (99.6%)	0.654	499 (96.7%)	493 (95.5%)	0.432	752 (70.2%)	751 (70.1%)	0.616
	Past Hx	0 (0.0%)	0 (0.0%)		0 (0.0%)	1 (0.2%)		32 (3.0%)	25 (2.3%)	
	Current	3 (0.6%)	2 (0.4%)		17 (3.3%)	22 (4.3%)		287 (26.8%)	295 (27.5%)	

Bold p-value indicates significant result at p < 0.05

*ETV* endoscopic third ventriculostomy, *IQR* interquartile range, *ASA* American Society of Anesthesiology

Analysis comparing preoperative characteristics of patients who underwent endoscopic third ventriculostomy or new shunt insertion after propensity score matching

For the ≤ 6 months age group, the match produced 477 patients per intervention group. The ETV group had longer operative times (68 vs 50 min, p < 0.0001), increased postoperative seizure (6.3% vs 0.4%, p < 0.001), increased related readmissions (15.7% vs 6.1%, p < 0.001), and increased related reoperations (14.7% vs 3.8%, p < 0.001). All other outcomes were similar.

For the 6 months to 2 years age group, the match produced 516 patients per intervention group. The ETV group had longer operative times (63 vs 54 min, p < 0.0001), increased organ space SSIs (2.5% vs 0.6%, p = 0.01), increased wound dehiscence (2.1% vs 0.2%, p = 0.004), increased postoperative seizure (3.3% vs 1.0%, p = 0.01), increased related readmissions (14.9% vs 7.8%, p < 0.001), and increased related reoperations (8.3% vs 3.7%, p = 0.002). All other outcomes were similar.

For the ≥ 2 years age group, the match produced 1071 patients per intervention group. The new shunt group had longer operative times (65 vs 59 min, p < 0.0001), increased total LOS (3 days IQR 2–8 vs 3 days IQR 1–6, p = 0.002), and increased mortality (1.5% vs 0.4%, p = 0.007). All other outcomes were similar. All post-match postoperative outcomes are shown in Table 3.

**Table 3:** Post-match postoperative outcomes

Variable	New Shunt	ETV	p-value	New Shunt	ETV	p-value	New Shunt	ETV	p-value
	≤ 6 months, n = 477 per group, n (%)			6 months—2 years, n = 516 per group, n (%)			≥ 2 years, n = 1071 per group, n (%)		
Operative Time, minutes (IQR)	50 (38–64)	68 (48–95)	** < 0.0001**	54 (43–72)	63 (42.5–91.5)	**0.0001**	65 (49–88)	59 (42–82)	** < 0.0001**
Length of total hospital stay, days (IQR)	3 (1–11)	3 (1–12.5)	0.641	2 (1–4)	2 (1–4)	0.350	3 (2–8)	3 (1–6)	**0.0019**
Still in Hospial > 30 Days	18 (3.77%)	28 (5.87%)	0.131	9 (1.74%)	4 (0.78%)	0.163	5 (0.47%)	7 (0.65%)	0.563
Death in 30 Days	6 (1.26%)	1 (0.21%)	0.058	3 (0.58%)	2 (0.39%)	0.654	16 (1.49%)	4 (0.37%)	**0.007**
Superficial Incisional SSI	2 (0.42%)	2 (0.42%)	1	3 (0.58%)	8 (1.55%)	0.13	8 (0.75%)	7 (0.65%)	0.796
Deep Incisional SSI	3 (0.63%)	0 (0.00%)	0.083	No cases	No cases		No cases	No cases	
Organ/Space SSI	6 (1.26%)	8 (1.68%)	0.59	3 (0.58%)	13 (2.52%)	**0.012**	12 (1.12%)	13 (1.21%)	0.841
Deep Wound Disruption/Dehiscence Occurrences	3 (0.63%)	9 (1.89%)	0.081	1 (0.19%)	11 (2.13%)	**0.004**	6 (0.56%)	14 (1.31%)	0.072
Pneumonia	1 (0.21%)	0 (0.00%)	0.317	2 (0.39%)	1 (0.19%)	0.563	2 (0.19%)	3 (0.28%)	0.654
Unplanned Intubation	7 (1.47%)	14 (2.94%)	0.122	1 (0.19%)	5 (0.97%)	0.101	8 (0.75%)	14 (1.31%)	0.199
On Ventilator > 48 Hours	1 (0.21%)	5 (1.05%)	0.214	No cases	No cases		No cases	No cases	
Progressive Renal Insufficiency	No cases	No cases		No cases	No cases		0 (0.00%)	1 (0.09%)	0.317
Acute Renal Fail	No cases	No cases		No cases	No cases		No cases	No cases	
Urinary Tract Infection	4 (0.84%)	4 (0.84%)	1	2 (0.39%)	1 (0.19%)	0.563	4 (0.37%)	5 (0.47%)	0.738
Coma > 24 Hours	No cases	No cases		No cases	No cases		No cases	No cases	
CVA/Stroke or Intracranial Hemorrhage	No cases	No cases		No cases	No cases		No cases	No cases	
Seizure Disorder	2 (0.42%)	30 (6.29%)	** < 0.001**	5 (0.97%)	17 (3.29%)	**0.01**	13 (1.21%)	14 (1.31%)	0.846
Nerve Injury	No cases	No cases		No cases	No cases		No cases	No cases	
IVH Grade 1	No cases	No cases		No cases	No cases		0 (0.00%)	1 (0.09%)	0.317
IVH Grade 2	No cases	No cases		No cases	No cases		No cases	No cases	
IVH Grade 3	No cases	No cases		No cases	No cases		No cases	No cases	
IVH Grade 4	No cases	No cases		No cases	No cases		No cases	No cases	
IVH Grade Unknown	2 (0.42%)	3 (0.63%)	0.654	1 (0.19%)	0 (0.00)	0.317	No cases	No cases	
Cardiac Arrest Requiring CPR	4 (0.84%)	2 (0.42%)	0.413	2 (0.39%)	1 (0.19%)	0.563	0 (0.00%)	3 (0.28%)	0.083
Transfusion ≥ 25 cc/kg	13 (2.73%)	18 (3.77%)	0.361	6 (1.16%)	6 (1.16%)	1	6 (0.56%)	5 (0.47%)	0.762
Ventricular Tachycardia	0 (0.00%)	1 (0.21%)	0.317	1 (0.19%)	3 (0.58%)	0.316	3 (0.28%)	6 (0.56%)	0.316
Sepsis	5 (1.05%)	6 (1.26%)	0.762	7 (1.36%)	10 (1.94%)	0.463	6 (0.56%)	7 (0.65%)	0.781
Septic Shock	No cases	No cases		1 (0.19%)	1 (0.19%)	1	1 (0.09%)	0 (0.00)	0.317
Central Line Infection	No cases	No cases		0 (0.00)	1 (0.19%)	0.317	No cases	No cases	
Related Readmission	29 (6.08%)	75 (15.72%)	** < 0.001**	40 (7.75%)	77 (14.92%)	** < 0.001**	90 (8.40%)	110 (10.27%)	0.137
Related Reoperation	18 (3.77%)	70 (14.68%)	** < 0.001**	19 (3.68%)	43 (8.33%)	**0.002**	65 (6.07%)	80 (7.47%)	0.197

Bold p-value indicates significant result at p < 0.05

*ETV* endoscopic third ventriculostomy, *IQR* interquartile range

Analysis comparing postoperative outcomes of patients who underwent endoscopic third ventriculostomy or new shunt insertion after propensity score matching

### Survival analysis for readmission

**Fig. 2:**
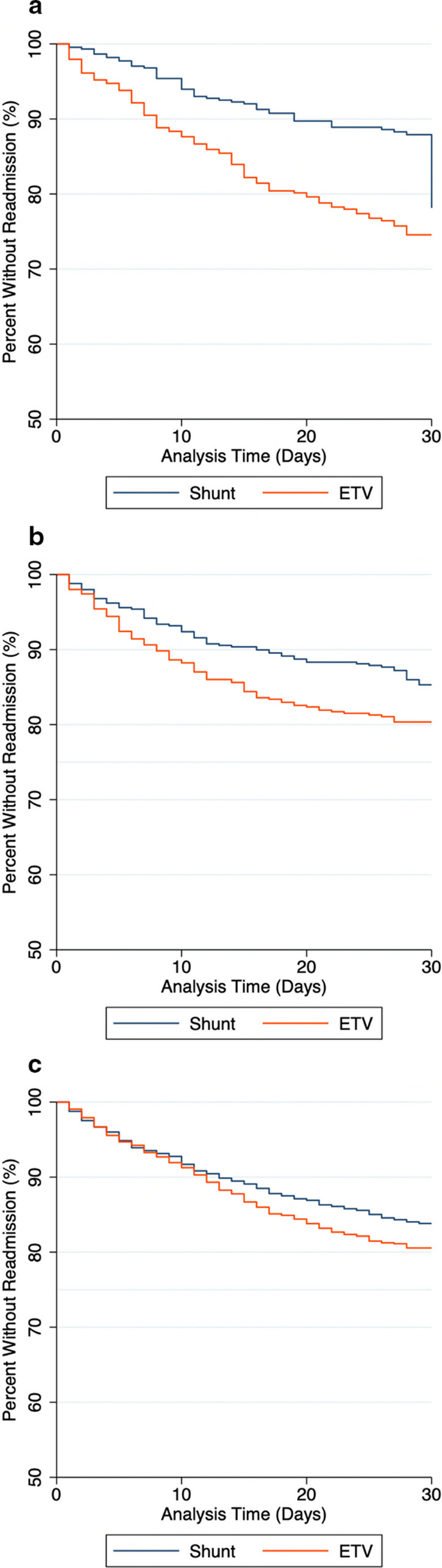
Survival Estimates for Readmission. Kaplan–Meier survival estimates for related readmission in each age group. There were significant differences in readmission survival for both the (**a**) ≤ 6 months age group (p < 0.001) and (**b**) 6 months to 2 years age group (p = 0.017), however no difference was seen in the (**c**) ≥ 2 years age group (p = 0.054)

Because of variable LOS, cases were subject to variable periods of risk for readmission during the first postoperative 30 days. Therefore, readmission was subjected to survival analysis. Cases that remained in the hospital after 30 days were excluded from this analysis as they were not at risk for readmission within this timeframe. There were significant differences in readmission for both the ≤ 6 months age group (p < 0.001) and 6 months to 2 years age group (p = 0.017), but no difference was seen in the ≥ 2 years age group (p = 0.054). Kaplan–Meier survival curves are shown in Fig. 2 for each age group.

## Discussion

Thirty-day surgical outcomes are the foundation of NSQIP for both children and adults, and they are a pillar of the US News and World Report hospital rankings [26]. They are relatively simple to collect, and as a quality measure they are responsive to process improvement. In the management of hydrocephalus they have been studied extensively not only in NSQIP but also in national and international multicenter registries [27, 28].

Comparisons of ETV and shunt insertion are challenging. Shunt insertion is undertaken with the expectation that hydrocephalus will be a chronic and incurable condition requiring life-long surgical maintenance, while ETV carries the potential for cure. Furthermore, although any case of hydrocephalus can, in principle, be treated by CSF shunt insertion, suitability for attempted treatment by ETV depends on clinical and anatomic factors, as exemplified in the ETV Success Score [23]. We have attempted to circumvent these challenges by using propensity score matching to compare cohorts of cases that, within the limits of the methodology, might have been treated by either procedure. There have been a number of prospective studies comparing ETV to shunt [8, 12]. Kulkarni et al. reported results from an international multi-institutional study including 27 centers. They found the actuarial success rates of ETV and shunt at 3 months to be 68% and 95%, respectively [12]. In our study, these rates were found to be 83% and 95% for the under 6 months age group, respectively. Similar to our study, they found that patients younger than 6 months do worse with ETV compared to shunt placement [12]. Uche et al. used data from a single institution which was comprised of age-matched patients with a mean age of 2.3 years and mean follow-up of 1.27 years [8]. Also similar to our study, they found ETV to be associated with decreased mortality in older children [8]. They also found increased rates of sepsis with shunt placement but earlier motor milestone achievement when compared to ETV [8].

A retrospective multi-institutional study including 41 different hospitals found ETV and shunt failure rates of 64.5% and 39.6%, respectively, at 1 year [13]. The infants in this study had a median age of 37 days (IQR 11–122 days) [13]. Even after adjustment for prematurity, IVH, and spina bifida, they still noted a higher risk of failure with ETV [13]. Likewise, our study also found increased failure rates with ETV in younger children compared to shunt. Another retrospective study of a single institution found no significant differences in outcomes at 1 year follow-up when comparing ETV and shunt, though this study may have been limited by low sample size [18].

Several case series have also compared ETV and shunt performance [16, 28]. El-Ghandour found that there was lower morbidity, mortality, and procedure failure associated with ETV compared to shunt placement [16]. de Ribaupierre et al. found a lower rate of ETV failure at 5-year follow-up but was unable to assess for statistical differences [28].

Only Kulkarni et al. have used propensity scores to adjust for unbalanced patient characteristics accounting for age and etiology of hydrocephalus in an international pediatric cohort [30]. They found the relative risk of ETV failure to be higher than shunt failure for the first 3 months, after which point risks becomes progressively lower for ETV [30]. The current study complements the Kulkarni report by drawing on a much larger sample size reflecting practices at a much larger number of institutions that are not necessarily engaged in hydrocephalus clinical research. In the context of the pediatric neurosurgical consensus about the long-term clinical superiority of ETV, this work demonstrates that what is good for 30-day outcomes is not necessarily good for patients. Assessment of quality in the management of childhood hydrocephalus requires a more discriminating approach than measurement of 30-day outcomes.

The short-term outcomes of ETV in younger age groups are unfavorable compared to CSF shunts, while the life-long advantages of successful ETV are compelling. These findings illustrate the inadequacy of existing quality metrics in the care of children with hydrocephalus. The ideal metric would be meaningful to families and payers, amenable to risk adjustment and other research analytics, and responsive to quality improvement measures. It would be strategic instead of tactical, assessing the value of the management of the entire course of a chronic illness like hydrocephalus instead of the success or failure of individual encounters. Beyond 30-day outcomes most clinical hydrocephalus research has employed survival analysis to study times to reoperation. This methodology is encounter-based, incomprehensible to many patients and families, useless to payers, and slow to detect improvements in care. At the senior author’s institution, an electronic medical record engineer has developed a filter algorithm for identifying patients in active follow-up (personal communication, Deborah Burchett). This denominator permits calculation of the fraction of patients requiring surgery in the preceding 12 months and number of hospital days required for those admissions. Reported on a monthly basis, these metrics are responsive to practice change. For parents, employers, and payers they reflect the annual probability that a child beneficiary will require surgery and the expected number of days for that child to spend in the hospital and for a parent to miss work. Sharing of the filter algorithm among children’s medical centers would open the way for risk adjustment and inter-institutional comparisons. Apropos of the current study, these metrics reward successful selection of patients for ETV. Denominator-based metrics from the electronic medical record may represent the future of quality measurement in the surgical management of hydrocephalus.

### Limitations

NSQIP-P only captures 30-day outcomes although it is known that ETV and shunt placement have significant failure rates outside of this period. NSQIP-P captures the care received at a select group of facilities that have made an expensive institutional commitment to quality improvement and may not reflect nationwide experiences. Disturbing disparities in surgical hydrocephalus outcomes have been reported between the research literature and population-based data [31], but the NSQIP sample is likely more broadly representative than much of the work that has been published in this area from institutions actively engaged in hydrocephalus research. The NSQIP-P PUF does not provide any unique hospital identifiers, so there can be no statistical accounting for clustering by facility. Actual neurosurgical decisions are based on clinical and anatomic factors other than the covariates entered into the propensity matching, so it is likely that not all patients included in the match were actually candidates for both procedures. The etiology of hydrocephalus is not recorded explicitly in NSQIP-P, and the reliability of extrapolation from associated diagnoses and comorbidities is uncertain. Finally, no imaging data are available.

## Conclusions

After stratification by age group, 30-day outcomes appear to depend on the age of the patient at the time of the index procedure. Among children < 2 years of age, short term outcomes after ETV were worse than after shunt insertion, but the opposite was true among older children. 30-day surgical outcomes unqualified by indications and contraindications for ETV lack face validity as a global quality measure in the management of childhood hydrocephalus. Lastly, neurodevelopmental outcomes are of much greater importance in the management of hydrocephalus than 30-day surgical complication rates, and no meaningful long-term comparative data are available at this time. A multicenter randomized controlled trial comparing early childhood neurodevelopmental outcomes after ETV and shunt insertion is underway [32].

## Data Availability

No datasets were generated or analysed during the current study.

## Abbreviations

ASA: American Society of Anesthesiology
CPR: Cardiopulmonary Resuscitation
CPT: Current Procedural Terminology
ETV: Endoscopic Third Ventriculostomy
IQR: Interquartile Range
IVH: Intraventricular Hemorrhage
LOS: Length of Stay
NSQIP-P: National Surgical Quality Improvement Program-Pediatric
SIRS: Systemic Inflammatory Response Syndrome
SSI: Surgical Site Infection

## Declarations

American College of Surgeons National Surgical Quality Improvement Program and the hospitals participating in the ACS NSQIP are the source of the data used herein; they have not verified and are not responsible for the statistical validity of the data analysis or the conclusions derived by the authors.

## References

[1] Drake JM, Kulkarni AV, Kestle J (2009) Endoscopic third ventriculostomy versus ventriculoperitoneal shunt in pediatric patients: a decision analysis. Childs Nerv Syst 25(4):467–472. 10.1007/s00381-008-0761-y 19139908 10.1007/s00381-008-0761-y

[2] Azab WA, Mijalcic RM, Nakhi SB, Mohammad MH (2016) Ventricular volume and neurocognitive outcome after endoscopic third ventriculostomy: is shunting a better option? A review. Childs Nerv Syst 32(5):775–780. 10.1007/s00381-016-3032-3 26861009 10.1007/s00381-016-3032-3

[3] Rasul FT, Marcus HJ, Toma AK, Thorne L, Watkins LD (2013) Is endoscopic third ventriculostomy superior to shunts in patients with non-communicating hydrocephalus? A systematic review and meta-analysis of the evidence. Acta Neurochir (Wien) 155(5):883–889. 10.1007/s00701-013-1657-5 23456239 10.1007/s00701-013-1657-5

[4] Dhandapani M, Yagnick NS, Mohanty M, Ahuja CK, Dhandapani S (2021) Clinical outcome, cognitive function, and quality of life after endoscopic third ventriculostomy versus ventriculo-peritoneal shunt in non-tumor hydrocephalus. Neurol India 69(Supplement):S556–S560. 10.4103/0028-3886.332271 35103013 10.4103/0028-3886.332271

[5] Bauer DF, Baird LC, Klimo P et al (2020) Congress of neurological surgeons systematic review and evidence-based guidelines on the treatment of pediatric hydrocephalus: Update of the 2014 guidelines. Neurosurgery 87(6):1071–1075. 10.1093/neuros/nyaa434 34791462 10.1093/neuros/nyaa434

[6] Kulkarni AV, Shams I, Cochrane DD, McNeely PD (2010) Quality of life after endoscopic third ventriculostomy and cerebrospinal fluid shunting: an adjusted multivariable analysis in a large cohort. J Neurosurg Pediatr 6(1):11–16. 10.3171/2010.3.PEDS09358 20593981 10.3171/2010.3.PEDS09358

[7] Kulkarni AV, Sgouros S, Leitner Y, Constantini S; International Infant Hydrocephalus Study Investigators. (2018) International Infant Hydrocephalus Study (IIHS): 5-year health outcome results of a prospective, multicenter comparison of endoscopic third ventriculostomy (ETV) and shunt for infant hydrocephalus. Childs Nerv Syst 34(12):2391–2397. 10.1007/s00381-018-3896-5 10.1007/s00381-018-3896-5 29987375

[8] Uche EO, Okorie C, Iloabachie I, Amuta DS, Uche NJ (2018) Endoscopic third ventriculostomy (ETV) and ventriculoperitoneal shunt (VPS) in non-communicating hydrocephalus (NCH): comparison of outcome profiles in Nigerian children. Childs Nerv Syst 34(9):1683–1689. 10.1007/s00381-018-3848-0 29860541 10.1007/s00381-018-3848-0

[9] Bogaczyk V, Fleck S, Berneiser J et al. (2022) Long-term quality of life after ETV or ETV with consecutive VP shunt placement in hydrocephalic pediatric patients [published correction appears in Childs Nerv Syst 38(10):1895. 10.1007/s00381-022-05657-7]. *Childs Nerv Syst*. 2022;38(10):1885–1894. 10.1007/s00381-022-05590-9 10.1007/s00381-022-05590-9 PMC9522746 35790573

[10] Stovell MG, Zakaria R, Ellenbogen JR et al (2016) Long-term follow-up of endoscopic third ventriculostomy performed in the pediatric population. J Neurosurg Pediatr 17(6):734–738. 10.3171/2015.11.PEDS15212 26870897 10.3171/2015.11.PEDS15212

[11] Takahashi Y (2006) Long-term outcome and neurologic development after endoscopic third ventriculostomy versus shunting during infancy. Childs Nerv Syst 22(12):1591–1602. 10.1007/s00381-006-0190-8 17021728 10.1007/s00381-006-0190-8

[12] Kulkarni AV, Sgouros S, Constantini S; IIHS Investigators (2016) International Infant Hydrocephalus Study: initial results of a prospective, multicenter comparison of endoscopic third ventriculostomy (ETV) and shunt for infant hydrocephalus. Childs Nerv Syst 32(6):1039–1048. 10.1007/s00381-016-3095-1 10.1007/s00381-016-3095-1 27107887

[13] Jernigan SC, Berry JG, Graham DA, Goumnerova L (2014) The comparative effectiveness of ventricular shunt placement versus endoscopic third ventriculostomy for initial treatment of hydrocephalus in infants. J Neurosurg Pediatr 13(3):295–300. 10.3171/2013.11.PEDS13138 24404970 10.3171/2013.11.PEDS13138

[14] Kulkarni AV, Drake JM, Kestle JR,et al. (2010) Predicting who will benefit from endoscopic third ventriculostomy compared with shunt insertion in childhood hydrocephalus using the ETV Success Score [published correction appears in J Neurosurg Pediatr. 2011 Feb;7(2):221] [published correction appears in J Neurosurg Pediatr. 2011 Feb;7(2):221. 10.3171/2010.8.PEDS103a]. J Neurosurg Pediatr 6(4):310–315. 10.3171/2010.8.PEDS103 10.3171/2010.8.PEDS103 20887100

[15] Lam S, Harris D, Rocque BG, Ham SA (2014) Pediatric endoscopic third ventriculostomy: a population-based study. J Neurosurg Pediatr 14(5):455–464. 10.3171/2014.8.PEDS13680 25238625 10.3171/2014.8.PEDS13680

[16] El-Ghandour NM (2011) Endoscopic third ventriculostomy versus ventriculoperitoneal shunt in the treatment of obstructive hydrocephalus due to posterior fossa tumors in children. Childs Nerv Syst 27(1):117–126. 10.1007/s00381-010-1263-2 20737274 10.1007/s00381-010-1263-2

[17] Lipina R, Reguli S, Dolezilová V, Kuncíková M, Podesvová H (2008) Endoscopic third ventriculostomy for obstructive hydrocephalus in children younger than 6 months of age: is it a first-choice method? Childs Nerv Syst 24(9):1021–1027. 10.1007/s00381-008-0616-6 18343929 10.1007/s00381-008-0616-6

[18] Prajapati HP, Ansari MA, Jaiswal M (2022) Comparative outcome analysis of endoscopic third ventriculostomy and ventriculoperitoneal shunt surgery in pediatric hydrocephalus: an experience of a tertiary care center. Asian J Neurosurg 17(2):227–234. Published 2022 Aug 26. 10.1055/s-0042-1750780 10.1055/s-0042-1750780 PMC9473859 36120619

[19] Greuter L, Schenker T, Guzman R, Soleman J (2024) Endoscopic third ventriculostomy compared to ventriculoperitoneal shunt as treatment for idiopathic normal pressure hydrocephalus: a systematic review and meta-analysis. Br J Neurosurg 38(6):1276–1282. 10.1080/02688697.2022.2149697 36537195 10.1080/02688697.2022.2149697

[20] Jesuyajolu DA, Zubair A, Nicholas AK, et al. (2022) Endoscopic third ventriculostomy versus ventriculoperitoneal shunt insertion for the management of pediatric hydrocephalus in African centers - A systematic review and meta-analysis. Surg Neurol Int 13:467. Published 2022 Oct 14. 10.25259/SNI_747_2022 10.25259/SNI_747_2022 PMC9610522 36324983

[21] Texakalidis P, Tora MS, Wetzel JS, Chern JJ (2019) Endoscopic third ventriculostomy versus shunt for pediatric hydrocephalus: a systematic literature review and meta-analysis. Childs Nerv Syst 35(8):1283–1293. 10.1007/s00381-019-04203-2 31129704 10.1007/s00381-019-04203-2

[22] Kulkarni AV, Sgouros S, Constantini S; International Infant Hydrocephalus Study Investigators. (2017) Outcome of treatment after failed endoscopic third ventriculostomy (ETV) in infants with aqueductal stenosis: results from the International Infant Hydrocephalus Study (IIHS). Childs Nerv Syst 33(5):747–752. 10.1007/s00381-017-3382-5 10.1007/s00381-017-3382-5 28357554

[23] Kulkarni AV, Riva-Cambrin J, Holubkov R et al (2016) Endoscopic third ventriculostomy in children: prospective, multicenter results from the Hydrocephalus Clinical Research Network. J Neurosurg Pediatr 18(4):423–429. 10.3171/2016.4.PEDS163 27258593 10.3171/2016.4.PEDS163

[24] Limbrick DD Jr, Baird LC, Klimo P Jr, Riva-Cambrin J, Flannery AM; Pediatric Hydrocephalus Systematic Review and Evidence-Based Guidelines Task Force. (2014) Pediatric hydrocephalus: systematic literature review and evidence-based guidelines. Part 4: Cerebrospinal fluid shunt or endoscopic third ventriculostomy for the treatment of hydrocephalus in children. J Neurosurg Pediatr 14 Suppl 1:30–34. 10.3171/2014.7.PEDS14324 10.3171/2014.7.PEDS14324 25988780

[25] Castro P, Piatt J (2024) Thirty-day outcomes of surgery for hydrocephalus: metrics in a large cohort from the National Surgical Quality Improvement Program-Pediatric. J Neurosurg Pediatr 34(5):438–451. Published 2024 Aug 23. 10.3171/2024.6.PEDS24183 10.3171/2024.6.PEDS24183 39178479

[26] Shaikh CF, Munir MM, Woldesenbet S, et al. (2024) Association of persistent poverty and U.S. News and World Report hospital rankings among patients undergoing major surgery. Am J Surg 228:11–19. 10.1016/j.amjsurg.2023.08.003 10.1016/j.amjsurg.2023.08.003 37596185

[27] Piatt JH Jr (2014) Thirty-day outcomes of cerebrospinal fluid shunt surgery: data from the National Surgical Quality Improvement Program-Pediatrics. J Neurosurg Pediatr 14(2):179–183. 10.3171/2014.5.PEDS1421 24926972 10.3171/2014.5.PEDS1421

[28] Al-Tamimi YZ, Sinha P, Chumas PD et al (2014) Ventriculoperitoneal shunt 30-day failure rate: a retrospective international cohort study. Neurosurgery 74(1):29–34. 10.1227/NEU.0000000000000196 24089046 10.1227/NEU.0000000000000196

[29] de Ribaupierre S, Rilliet B, Vernet O, Regli L, Villemure JG (2007) Third ventriculostomy vs ventriculoperitoneal shunt in pediatric obstructive hydrocephalus: results from a Swiss series and literature review. Childs Nerv Syst 23(5):527–533. 10.1007/s00381-006-0283-4 17226034 10.1007/s00381-006-0283-4

[30] Kulkarni AV, Drake JM, Kestle JR et al (2010) Endoscopic third ventriculostomy vs cerebrospinal fluid shunt in the treatment of hydrocephalus in children: a propensity score-adjusted analysis. Neurosurgery 67(3):588–593. 10.1227/01.NEU.0000373199.79462.21 20647973 10.1227/01.NEU.0000373199.79462.21

[31] LeHanka A, Piatt J (2020) Readmission and reoperation for hydrocephalus: a population-based analysis across the spectrum of age. J Neurosurg 134(3):1210–1217. Published 2020 May 29. 10.3171/2020.3.JNS20528 10.3171/2020.3.JNS20528 32470941

[32] ClinicalTrials.gov [Internet]. Bethesda (MD): National Library of Medicine (US); 2000– [cited 2024 Dec 18]. Identifier NCT04177914, A Prospective Study of Choroid Plexus Coagulation in Infants With Hydrocephalus. Available from: https://clinicaltrials.gov/study/NCT04177914. Last accessed 12-18-24.

